# Virus-like particle vaccine with authentic quaternary epitopes protects against Zika virus-induced viremia and testicular damage

**DOI:** 10.1128/jvi.02322-24

**Published:** 2025-02-27

**Authors:** Sandra R. Abbo, Kexin Yan, Corinne Geertsema, Tessy A. H. Hick, Jort J. Altenburg, Gwen Nowee, Chris van Toor, Jan W. van Lent, Eri Nakayama, Bing Tang, Stefan W. Metz, Ryan Bhowmik, Aravinda M. de Silva, Natalie A. Prow, Ricardo Correia, Paula M. Alves, António Roldão, Dirk E. Martens, Monique M. van Oers, Andreas Suhrbier, Gorben P. Pijlman

**Affiliations:** 1 Laboratory of Virology, Wageningen University & Research 4508 , Wageningen, the Netherlands; 2 Inflammation Biology Group, QIMR Berghofer Medical Research Institute, Brisbane, Australia; 3 Bioprocess Engineering, Wageningen University & Research 4508 , Wageningen, the Netherlands; 4 Department of Virology I, National Institute of Infectious Diseases, Tokyo, Japan; 5 Department of Microbiology and Immunology, University of North Carolina at Chapel Hill 318275 , Chapel Hill, North Carolina, USA; 6 IBET, Instituto de Biologia Experimental e Tecnológica 449442 , Oeiras, Portugal; 7 ITQB NOVA, Instituto de Tecnologia Química e Biológica António Xavier, Universidade Nova de Lisboa 98819 , Oeiras, Portugal; 8 GVN Centre of Excellence, Australian Infectious Disease Research Centre, Brisbane, Queensland, Australia; St. Jude Children's Research Hospital, Memphis, Tennessee, USA

**Keywords:** Zika virus, vaccines, virus-like particles, insect cells, quaternary epitopes

## Abstract

Zika virus (ZIKV) caused unprecedented outbreaks in South America and the Caribbean in 2015–2016, leading primarily to a series of abnormalities in neonates termed congenital Zika syndrome. The threat of ZIKV reemergence has seen the development of multiple ZIKV vaccines that are at the preclinical stage or in early-stage clinical trials. Herein, we describe a pathway to the development of ZIKV vaccines generated using a baculovirus-insect cell expression system, which is widely applied for the manufacture of biologics for human use. Virus-like particle (VLP) vaccines comprising CprME and subviral particle (SVP) vaccines comprising prME were evaluated for their ability to mediate protection against ZIKV challenge in *Ifnar1*^−/−^ mice. Initial attempts resulted in VLP and SVP vaccines that failed to present quaternary epitopes and did not provide effective protection. To improve the SVP vaccine, two modifications were introduced: firstly, an alanine to cysteine substitution (A264C) in the E domain II region to promote the formation of stabilized E homodimers and, secondly, the use of *Spodoptera frugiperda* Sf9 insect cells that had been adapted to grow and produce vaccine at a neutral pH of 7. E homodimers largely retain their pre-fusion conformation at pH 7, which is a requirement for the induction of effective neutralizing antibody responses. The stabilized SVP-A26C vaccine induced high levels of neutralizing antibodies and protected male *Ifnar1*^−/−^ mice against viremia and testicular damage. Our study reiterates the need to present the immune system with E dimers arranged in authentic quaternary conformations and provides a scalable production method for this novel ZIKV vaccine.

**IMPORTANCE:**

We describe the generation of a subviral particle (SVP) vaccine comprising prME proteins of ZIKV, with an envelope protein substitution, A264C, that stabilizes E dimer formation. The SVP vaccine was produced in a novel Sf9 insect cell line adapted to grow in suspension at pH 7. The study highlights the importance of challenge experiments to ascertain whether the responses induced by an experimental vaccine actually mediate protection against virus infection and disease. The study also reiterates the contention that effective flavivirus vaccines need to present the immunogen in an authentic tertiary and quaternary structure with a pre-fusion conformation.

## INTRODUCTION

Zika virus (ZIKV) is a mosquito-borne pathogen that caused an explosive outbreak of human disease primarily in the Americas during 2015 and 2016 (1, 2), although transmission was also reported in a range of countries worldwide (3). The key disease manifestation, congenital Zika syndrome (CZS), represents a spectrum of congenital malformations in newborns, including but not limited to microcephaly (4). Around 4,000 children in 27 countries have been affected, with the largest number of cases occurring in Brazil (5). CZS is often associated with developmental delays and neurologic sequelae in infants (6) and can lead to a series of chronic health issues (7, 8) including increased vulnerability to infectious and respiratory conditions (9). The burden of congenital anomalies, nervous system disorders, and infectious diseases also contribute to an increase in mortality in children live-born with CZS (10). ZIKV infection in adults can cause Guillain-Barré syndrome (11), with ZIKV also able to infect the male reproductive tract (12), where it can persist for extended periods (13, 14).

In 2022, the World Health Organization (WHO) declared the Global Arbovirus Initiative against arboviruses, which includes ZIKV (15). The initiative seeks to strengthen global preparedness to future arboviral disease outbreaks, which involves *inter alia* development of new vaccines and vaccine technologies. A number of ZIKV vaccines are in development and use a range of modalities (16–19), with a paramount consideration being safety in pregnant women and women of childbearing age (20). Long-term protective immunity is also desirable (21), given that ZIKV outbreaks are likely again to occur in resource-poor countries. Although mRNA vaccines have seen spectacular advances in recent years and have been applied to ZIKV vaccine design (18), lack of long-term protective immunity remains an issue (19, 22).

ZIKV belongs to the species *Orthoflavivirus zikaense*, genus *Orthoflavivirus* in the family *Flaviviridae*, and contains a positive-sense, single-stranded 11 kilobase (kb) RNA genome. ZIKV has three structural proteins: capsid (C), precursor membrane (prM), and envelope (E) that together build a spherical virus particle of ~50 nm in diameter. Immature ZIKV particles bud into the endoplasmic reticulum (ER) lumen and travel through the Golgi apparatus to the cell surface. During this process, conformational changes in the E glycoprotein and cleavage of prM in the precursor peptide (pr) and M protein occur. Dissociation of pr upon egress into the extracellular environment results in mature, smooth particles displaying 90 E homodimers on their surface (23–26). The highly ordered, tertiary and quaternary structures adopted by the antiparallel E homodimers on the virion particle surface (27) represent the main target of neutralizing antibodies, with presentation of authentic structures to B cells likely important for vaccine-mediated generation of protective neutralizing antibody responses (19, 24, 28, 29).

Herein, we evaluate the prototype vaccines against ZIKV generated using the baculovirus-insect cell expression system that comprise virus-like particle (VLP) or subviral particle (SVP) vaccines. The VLP vaccine was produced by expressing the ZIKV structural proteins C, prM, and E, which self-assemble into particles that are structurally similar to wild-type virus. SVP vaccines were produced by expression and self-assembly of only both prM and E proteins. VLP and SVP vaccines lack a viral genome and are unable to replicate, with replication-incompetent ZIKV vaccines generally viewed as safer for pregnant women (30). The vaccines were evaluated in established *Ifnar1*^−/−^ mouse models of ZIKV infection and disease (24, 31).

## MATERIALS AND METHODS

### Regulatory compliance

Breeding and use of GM mice were approved under a Notifiable Low Risk Dealing (NLRD) Identifier: NLRD_Suhrbier_Oct2020: NLRD 1.1(a). Agistment conditions were as follows: light = 12:12 hour dark/light cycle, 7:45 a.m. sunrise and 7:45 p.m. sunset, 15 minute light dark and dark light ramping time; enclosures, M.I.C.E cage (Animal Care Systems, Colorado, USA); ventilation, 100% fresh air, eight complete air exchange/h/room; in-house enrichment, paper cups (Impact-Australia), tissue paper, cardboard rolls; bedding, PuraChips (Able scientific) (aspen fine); food, double bagged norco rat and mouse pellet (AIRR, Darra, QLD); water, deionized water acidified with HCl (pH = 3.2); and temperature, 22 ± 1°C (32).

### Cell culture

*Spodoptera frugiperda* Sf21 (Gibco, Carlsbad, CA, USA), Sf9 (Gibco), and Sf9-ET (33) cells were grown at 27°C. Monolayers of Sf21 cells were cultured in Grace’s medium (Gibco) supplemented with 10% fetal bovine serum (FBS; Gibco). Monolayers of Sf9-ET cells were grown in Sf900II medium (Gibco) containing 5% FBS and 100 µg/mL geneticin (Gibco). Monolayers and suspension cultures of Sf9 cells were maintained in Sf900II serum-free medium supplemented with 50 µg/mL gentamycin (Gibco). The African green monkey kidney Vero cell line was grown in RPMI 1640 medium supplemented with 10% FBS at 37°C and 5% CO_2_.

### Insect cell adaptation to neutral pH

Sf9 suspension cultures (2 × 10^6^ cells/mL at day 0) were grown in culture medium of uncontrolled pH or culture medium set to pH 6.6, 6.8, or 7.0. Culture medium was set to the desired pH using 0.5 M NaOH at day 0 and checked each day thereafter with a pH monitoring probe to ensure a constant pH (34). Cell concentration and cell viability were determined daily using a Countess II Automated Cell Counter (Invitrogen) according to supplied protocol. Adaptation of Sf9 cells to higher culture pH by an adaptive laboratory evolution approach was performed using a stepwise approach (i.e., adaptation from the standard culture pH of 6.2 to, initially, pH of 6.5, then 6.8, and finally 7.0) as described (35). Cells were cultured in medium containing a 1:1 mixture of Sf900 II (Gibco) medium and a solution composed of 50 mM HEPES, 124 mM sucrose, 5 mM glucose, 50 mM NaCl, 20 mM KCl, 3 mM CaCl_2_, 10 mM MgSO4, and 0.1% (w/v) Pluronic F-68; pH was adjusted to 6.5, 6.8, or 7.0 by adding 1 M NaOH and sterile filtered using a 0.22 µm Stericup (Millipore). Cells were subcultured in each pH step until a constant growth rate and cell viability over 95% were observed, and master cell banks were prepared after adaptation at each pH.

### Generation of recombinant baculoviruses

ZIKV structural cassettes CprME, prME, and EΔTM (secreted E; lacking a transmembrane domain) were amplified from the cDNA of the Asian lineage ZIKV Suriname 2016 isolate (NL00013, GenBank KU937936.1, isolated from a patient in The Netherlands [36] and obtained from the Erasmus Medical Center, Rotterdam, The Netherlands) by PCR using Phusion High-Fidelity DNA Polymerase (New England Biolabs, Ipswich, MA, USA) and a 2720 Thermal Cycler (Applied Biosystems). Primers (Table 1) contained attB recombination sites to enable Gateway cloning (Invitrogen). The ZIKV structural cassettes were recombined into a pDONR207 plasmid (Invitrogen) and subsequently into a pDEST8 plasmid (Invitrogen) downstream of the baculovirus polyhedrin promoter. The pDEST8 plasmid containing the prME cassette was used to create an alternative pDEST8 plasmid containing a prME cassette with an alanine to cysteine substitution (A264C) as previously described for the production of stable, covalently linked dengue virus (DENV) and ZIKV E homodimers (37–41). The A264C substitution was introduced by quick change PCR using primers described in Table 1. Next, the four cassettes (CprME, prME, prME-A264C, EΔTM) were transposed into the improved *Autographa californica* multiple capsid nucleopolyhedrovirus (AcMNPV) backbone BACe56 with a relocated attTn7 transgene insertion site (42). Sf21 cells were transfected with purified recombinant bacmid DNA using ExpreS^2^ TR (ExpreS^2^ion Biotechnologies). Recombinant baculovirus titers were determined in Sf9-ET cells and expressed as 50% tissue culture infectious dose per mL (TCID_50_/mL).

**TABLE 1:** Primers used in this study^*a*^

Target	Primer name	Primer sequence (5′→3′)	Product (kb)
ZIKV CprME	attB1-ZIKV-C-F	**GGGGACAAGTTTGTACAAAAAAGCAGGCTTA**ACCATGAAAAACCCAAAAAAGAAATC	2.4
	attB2-ZIKV-Estem/anchor-R	**GGGGACCACTTTGTACAAGAAAGCTGGGTA**TTAAGCAGAGACGGCTGTGGATA	
ZIKV prME	attB1-ZIKV-pr-F	**GGGGACAAGTTTGTACAAAAAAGCAGGCTTA**ACCATGGGCGCAGATACTAGTGTCGG	2.0
	attB2-ZIKV-Estem/anchor-R	**GGGGACCACTTTGTACAAGAAAGCTGGGTA**TTAAGCAGAGACGGCTGTGGATA	
ZIKV EΔTM	attB1-ZIKV-E-F	**GGGGACAAGTTTGTACAAAAAAGCAGGCTTA**ACCATGTCAACGAGCCAAAAAGTCAT	1.3
	attB2-6xHis-tag-ZIKV-E-R	**GGGGACCACTTTGTACAAGAAAGCTGGGTA**TTAGTGATGGTGATGGTGATGTTTTCCAATGGTGCTGCCAC	
pDEST8/ZIKV- prME- A264C	ZIKV-E-A264C-F	TCAAGAAGGATGCGTTCACACGGCCCTTGCTGG	8.5
	ZIKV-E-A264C-R	CCGTGTGAACGCATCCTTCTTGACTCCCTAGAA	

^*a*^ The attB site of each primer is shown in bold. The mutations used to create the A264C substitution are underlined.

### Production of ZIKV vaccines

For small-scale vaccine production, 8 × 10^6^ Sf21 or Sf9 insect cells were seeded as monolayers in 75 cm^2^ flasks. Cells were infected with recombinant baculovirus containing the ZIKV CprME structural cassette (BACe56/ZIKV-CprME), the ZIKV prME structural cassette (BACe56/ZIKV-prME), or the ZIKV prME-A264C structural cassette (BACe56/ZIKV-prME-A264C) for Zika VLP, Zika SVP, or Zika SVP-A264C vaccine production, respectively. Soluble ZIKV E subunit was produced by infecting cells with recombinant baculovirus harboring the structural cassette ZIKV EΔTM (BACe56/ZIKV-EΔTM). Uninfected cells as well as cells infected with recombinant baculovirus expressing a green fluorescent protein (BAC/GFP) (43) were used as negative controls. Cells were infected at a multiplicity of infection (MOI) of 10 TCID_50_ units per cell (CprME, prME, EΔTM, GFP) or 0.4 TCID_50_ units per cell (prME-A264C). After infection, cells were incubated at 27°C for 4 hours. Afterward, the cell culture medium was replaced by fresh medium, and cells were incubated at 27°C for 3–4 days.

For larger scale vaccine production, Sf9 suspension cultures containing 2.0–2.5 × 10^6^ cells/mL were infected with BACe56/ZIKV-CprME or BACe56/ZIKV-prME or BACe56/ZIKV-prME-A264C at an MOI of 0.01–5 TCID_50_ units per cell. Cells were incubated at 27°C for 3 days. For infections performed with neutral-pH-adapted cells at bioreactor scale, pH was monitored and controlled at 7.0 during the entire process using NaOH. Cells and medium were harvested and separated by centrifugation at 1,700 rpm for 5 minutes using a Heraeus Megafuge 40R centrifuge (Thermo Scientific). The cell pellet was resuspended in PBS, and the supernatant containing the Zika VLP and SVP vaccines was filtered through a 0.45 µm filter.

### Purification of ZIKV vaccines

First, 7% (w/v) polyethylene glycol (PEG)-6000 and 0.5 M NaCl were added to the filtered medium to precipitate the VLP/SVPs. After 2 hours at room temperature (RT) and following centrifugation at 4,700 rpm for 15 minutes using a Heraeus Megafuge 40R centrifuge (Thermo Scientific), the pellet was dissolved in GTNE buffer (200 mM glycine, 50 mM Tris/HCl, 100 mM NaCl, 1 mM EDTA, pH 7.3). The VLP/SVPs in GTNE were then loaded onto a 30%–80% (w/v) continuous sucrose gradient (prepared in GTNE) and subjected to centrifugation at 45,000 rpm for 2 hours using an SW55 rotor (Beckman). Twenty-five fractions were collected from the top of the gradient and analyzed for the presence of ZIKV E protein using Western blot. ZIKV E protein containing fractions was pooled and centrifuged again at 45,000 rpm for 2 hours. The pellet was then dissolved in GTNE buffer, and the pure VLP/SVPs were stored at −80°C. Samples were subsequently analyzed by Western blot to detect and quantify ZIKV E protein and by transmission electron microscopy to check the integrity of the particles.

### Zika VLP/SVP vaccine protein analysis and Western blot

ZIKV proteins from cell fractions, medium fractions, and purified VLP/SVP fractions were analyzed using sodium dodecyl sulfate polyacrylamide gel electrophoresis (SDS-PAGE) followed by Western blotting. Samples were run on a Mini-PROTEAN TGX gel (Bio-Rad), with a trans-blot semi-dry transfer cell (Bio-Rad) used to transfer the proteins to an Immobilon-P membrane (Merck Millipore). The membrane was blocked at 4°C overnight using 1% skim milk powder dissolved in PBS containing 0.05% Tween (PBS-T). The membrane was incubated at RT for 1 hour with pan-flavivirus α-E monoclonal antibody (mAb) 4G2 (44) diluted 1:1000 in 1% skim milk. After washing the membrane three times with PBS-T, alkaline phosphatase, conjugated goat anti-mouse IgG secondary antibody (Sigma-Aldrich) diluted 1:2500 in PBS-T was added. After 1 hour, the membrane was washed three times with PBS-T and subsequently incubated with alkaline phosphatase buffer as described (43) for 10 minutes. The membrane was developed using NBT/BCIP (Roche Diagnostics).

### Quantification of Zika VLP/SVP vaccines

The purified ZIKV vaccines were quantified using a dilution series of pure DENV serotype 4 E protein (The Native Antigen Company). Samples with purified Zika VLP/SVP and samples containing serial twofold dilutions of 3 µg DENV E were prepared and analyzed by SDS-PAGE and Western blot using the pan-flavivirus 4G2 mAb as described above. The intensity of protein bands was compared to estimate the concentration of Zika VLP/SVP in the purified fractions.

### Antibody ELISA, neutralization assays, and virus titration

IgG responses were measured by standard ELISA using whole ZIKV_MR766_ as antigen as described (45, 46). The neutralizing ability of mouse sera from vaccinated animals was also determined as described (45). Briefly, serum was heat-inactivated at 56°C for 30 minutes. Diluted serum was incubated with 100 TCID_50_ of ZIKV_Natal_ (GenBank KU527068) or ZIKV_PRVABC59_ (GenBank LC002520.1) for 2 hours, and Vero cells (10^5^ cells/ml) were added afterward. Cells were fixed at 7 days post infection and stained with crystal violet, after which the reciprocal 50% neutralization titers were determined. To validate this assay, sera from four mice immunized with UV-inactivated virus were tested and provided reciprocal anti-ZIKV_PRVABC59_ 50% neutralization titers of 916, 1556, 1847, and 1717. Serum viremia was measured by TCID_50_ assays as previously reported (47).

### Epitope display analysis

Display of epitopes on Zika VLP/SVP vaccines was analyzed by ELISA using a panel of well-defined mouse or human-derived mAbs targeting the flavivirus E protein (Table 2) (28, 37, 48, 49). ZIKV H/PF/2013 (GenBank KJ776791.2) wild-type virus and recombinant E subunit (37) were included for comparison. All analyses were carried out in duplicate. Zika VLP/SVP vaccines, wild-type ZIKV, and ZIKV E subunit were captured using 4G2 mAb (44) (for human detection antibodies) or 1M7 mAb (50) (for mouse antibodies). The DENV serotype 2 specific mAb 3H5 (51) was used as a negative control. Antibody binding was determined using alkaline phosphatase-conjugated antihuman or anti-mouse IgG secondary antibodies (Sigma) in combination with alkaline phosphatase substrate (Sigma). Absorbance was measured at 405 nm.

**TABLE 2:** Monoclonal antibodies (mAbs) used for epitope display analysis^*a*^

mAb	M/H	Binding	Neutralization (W/M/S)	E protein-binding region	Binding to DENV serotypes and ZIKV					Reference
					DV1	DV2	DV3	DV4	ZIKV	
4G2	M	F-CR	W	DII FL	++	++	+++	+++	+++	(44)
1M7	H	F-CR	M	DII FL	+++	++	+++	+++	+++	(50)
A11 (EDE2)	H	F-CR	DV:S ZIKV:W	DI/DII/DIII Q	+++	+++	+++	+++	+	(52)
B7 (EDE2)	H	F-CR	DV:S ZIKV:W	DI/DII/DIII Q	+++	+++	+++	+++	+	(52)
C8 (EDE1)	H	F-CR	DV:S ZIKV:S	DI/DII/DIII Q	+++	+++	+++	+++	++	(52)
C10 (EDE1)	H	F-CR	DV:S ZIKV:S	DI/DII/DIII Q	+++	+++	+++	+++	++	(52)
ZKA-64	H	ZIKV	ZIKV:S	DIII	–	–	–	–	+++	(53)
Z3L1	H	ZIKV	ZIKV:S	DI/DII	–	–	–	–	+++	(54)
Z23	H	ZIKV	ZIKV:S	DIII	–	–	–	–	+++	(54)
A9E	H	ZIKV	ZIKV:S	DI Q^*b*^	–	–	–	–	+++	(28)
G9E	H	ZIKV	ZIKV:S	DII Q^*b*^	–	–	–	–	+++	(28)
Z20	H	ZIKV	ZIKV:S	DII Q	–	–	–	–	+++	(54)
ZIKV-117	H	ZIKV	ZIKV:S	DII Q	–	–	–	–	+++	(55)
3H5	M	DV2	DV2:S	DIII LR	–	+++	–	–	–	(51)

^*a*^ A panel of characterized mouse (M) or human (H) derived mAbs was used to interrogate binding to Zika VLP/SVP vaccines. Abbreviations: EDE, E dimer epitope dependent; F-CR, flavivirus cross-reactive; W/M/S,weakly, moderately or strongly neutralizing; DI, DII, DIII, binding to E-domain I, II, or III; FL, fusion loop; LR, lateral ridge; Q, quaternary; –/+/++/+++, no/weak/moderate/strong binding.

^*b*^ Not completely mapped.

### Transmission electron microscopy

Purified Zika VLP/SVP vaccines in GTNE buffer were loaded onto 200 mesh carbon-coated copper grids (Electron Microscopy Sciences). After 2 minutes at RT, the excess liquid was removed, and 2% ammonium molybdate (pH 7) was added to the grids. After 30 seconds at RT, the excess liquid was again removed. After air-drying, the grids were analyzed using a JEOL JEM-1011 transmission electron microscope. VLP/SVP diameters were determined using ImageJ in combination with in-house macros.

### Zika VLP and SVP vaccination and challenge of female *Ifnar1*^−/−^ mice

Female interferon-α/β receptor knockout (*Ifnar1*^−/−^) mice (C57BL/6J background; ≈13 weeks old) were immunized with 1 µg Zika VLPs or SVPs per mouse (56). As a negative control, a group of female *Ifnar1*^−/−^ mice was vaccinated with 1 µg CHIKV VLPs, which were produced and purified as described (43, 57). The vaccines or PBS were administered once via the intramuscular route (40 µL into both quadriceps muscles). The mice were challenged by subcutaneous inoculation with 10^3^ TCID_50_ ZIKV_MR766_ (GenBank LC002520.1) 6 weeks after the first immunization or 10^4^ TCID_50_ ZIKV_Natal_ (GenBank KU527068) 8 weeks after the first immunization (45, 47). ZIKV_MR766_ infection is lethal in *Ifnar1*^−/−^ mice, with mice euthanized at ethically defined end points (58).

### Zika VLP and SVP vaccination and challenge of male *Ifnar1*^−/−^ mice

Male *Ifnar1*^−/−^ mice (≈12 weeks old) were immunized at three different times with 1 µg Zika VLPs or SVPs mixed in a 1:1 vol ratio with AddaVax adjuvant (InvivoGen) (59). Negative control groups were male *Ifnar1*^−/−^ mice vaccinated with CHIKV VLPs (43) formulated with AddaVax or inoculated with PBS. The vaccines or PBS were administered via the intramuscular route (40 µL into both quadriceps muscles). A positive control group was infected with 10^4^ TCID_50_ ZIKV_Natal_ s.c. at the base of the tail. Mice were challenged by subcutaneous inoculation with 10^3^ TCID_50_ ZIKV_PRVABC59_ (GenBank MH158237.1) 14 weeks after initial immunization (58).

### SVP and SVP-A264C (pH 7) vaccination and challenge of male *Ifnar1*^−/−^ mice

Male *Ifnar1*^−/−^ mice (≈12 weeks old) were immunized at three different times with 1 µg of the SVP-A264C (pH 7) or SVP (pH 7) vaccines mixed in a 1:1 vol ratio with AddaVax adjuvant. Negative control groups were male *Ifnar1*^−/−^ mice inoculated with PBS formulated with AddaVax or PBS alone. The positive control was male *Ifnar1*^−/−^ mice infected with ZIKV_Natal_ as above. Mice were challenged by subcutaneous inoculation with 10^3^ TCID_50_ ZIKV_PRVABC59_ (GenBank MH158237.1) 11 weeks after initial immunization (58)

### Statistics

The *t*-test was used if the difference in variances was <4 fold, skewness was >−2, and kurtosis was <2. The *t*-test significance and variance were determined using Microsoft Excel. Skewness and kurtosis were determined using IBM SPSS Statistics for Windows v19.0. Otherwise, the nonparametric Kolmogorov-Smirnov exact test was performed using GraphPad Prism 10.

## RESULTS

### Production and purification of Zika VLPs and SVPs from insect cells

**Fig 1:**
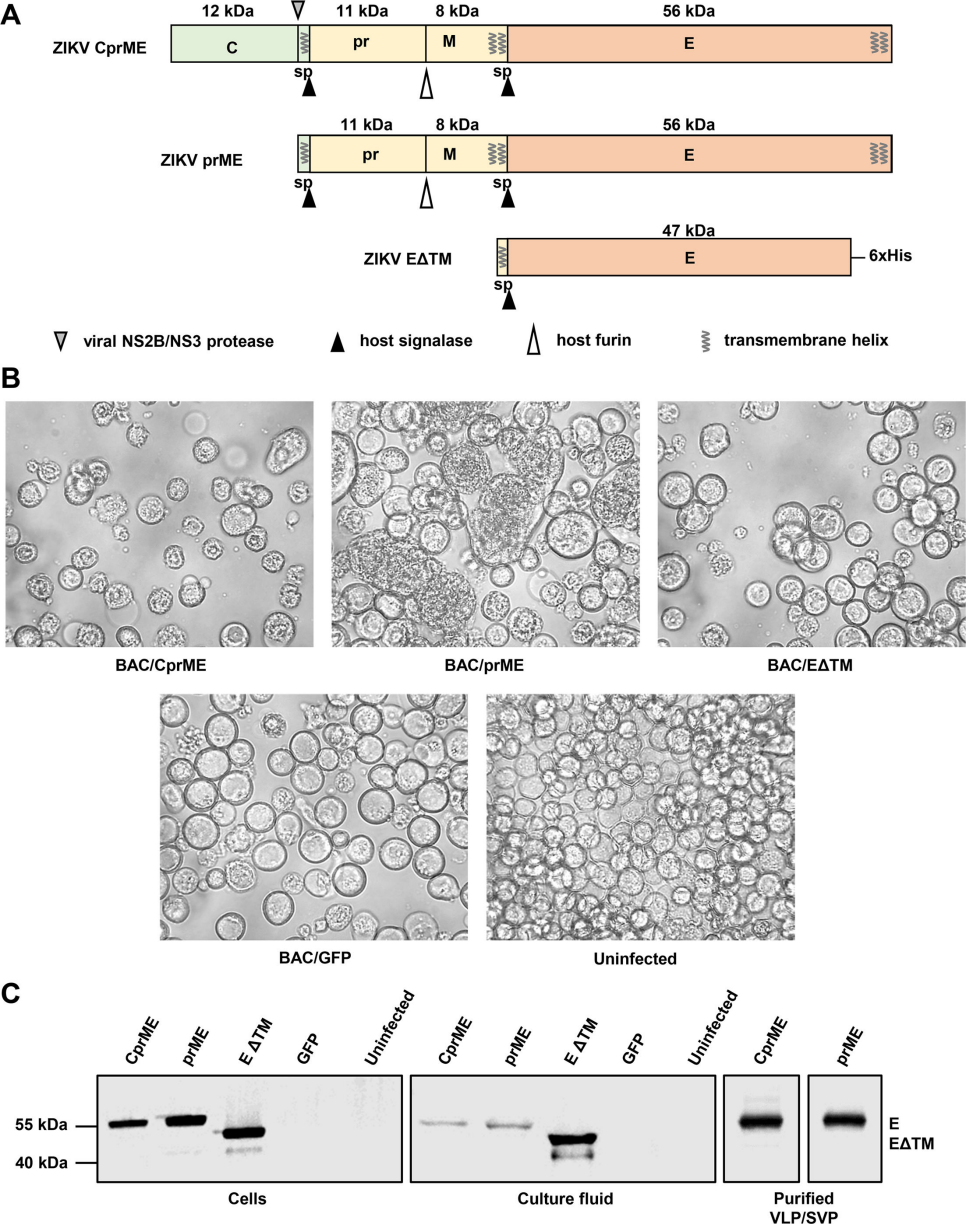
Production of Zika VLP and SVP vaccines using insect cells. (**A**) Schematic representation of the structural cassettes used for the production of Zika VLP (CprME), ZIKV SVP (prME) vaccines, and secreted ZIKV E subunit (EΔTM) in insect cells. The molecular mass of each viral protein is shown in kDa. Cleavage sites of viral protease, host signalase, and host furin are indicated, as well as predicted signal peptide (sp) sequences and transmembrane helices. ZIKV EΔTM contains a C-terminal histidine tag (6xHis). (**B**) Sf21 insect cells infected with the indicated baculoviruses at 4 days post infection or uninfected cells. (**C**) Western blot analysis of ZIKV E protein expression in Sf21 insect cells infected with recombinant baculoviruses containing the indicated cassettes at 4 days post infection, in culture fluids from those infected Sf21 cells, and in VLP/SVP vaccines purified by sucrose gradient. Western blotting used the pan-flavivirus anti-E mAb 4G2.

To produce Zika VLP or SVP vaccines in insect cells, recombinant baculoviruses expressing the structural cassette ZIKV CprME or prME (Fig. 1A), respectively, were constructed. A secreted ZIKV E subunit was produced for comparison by expressing the ZIKV E coding region without the C-terminal transmembrane domain (Fig. 1A and E ΔTM). The prM and E sequences contained their native signal peptides for translocation to the ER. Recombinant baculoviruses BACe56/ZIKV-CprME, BACe56/ZIKV-prME, and BACe56/ZIKV-EΔTM were used to infect Sf21 cells at an MOI of 10 (TCID_50_ per cell). Uninfected cells and cells infected with a recombinant baculovirus expressing GFP (BAC/GFP) (43) were included as negative controls. After 4 days, signs of baculovirus infection were observed for infections with BACe56/ZIKV-CprME, BACe56/ZIKV-prME, BACe56/ZIKV-EΔTM, and BAC/GFP (Fig. 1B). The infected cells showed an increased cell diameter, enlarged nuclei, detachment, growth arrest, and lysis. Uninfected cells did not show these effects (Fig. 1B). BACe56/ZIKV-prME-infected cells also showed formation of large syncytia (Fig. 1B, top center). The syncytia were most likely caused by fusogenic activity of the ZIKV E protein, which is also responsible for fusion of the viral envelope with the endosomal membrane during virus infection (60).

The baculovirus-infected cells and the culture fluid were analyzed by Western blot using the anti-E mAb 4G2. Expression of ZIKV CprME and ZIKV prME structural cassettes resulted in the detection of a protein at ∼55 kDa, similar to the predicted molecular mass of processed E protein (56 kDa, in both cell and medium fractions [Fig. 1C]). Expression of ZIKV-EΔTM showed a protein at ∼50 kDa (Fig. 1C), which corresponds to the predicted molecular mass of processed EΔTM (47 kDa). VLP/SVP vaccines were isolated from the culture fluid using PEG precipitation followed by 30%–80% continuous sucrose gradient purification. Purification was confirmed by Western blot analysis (Fig. 1C).

### Characterization of Zika VLP and SVP vaccines

**Fig 2:**
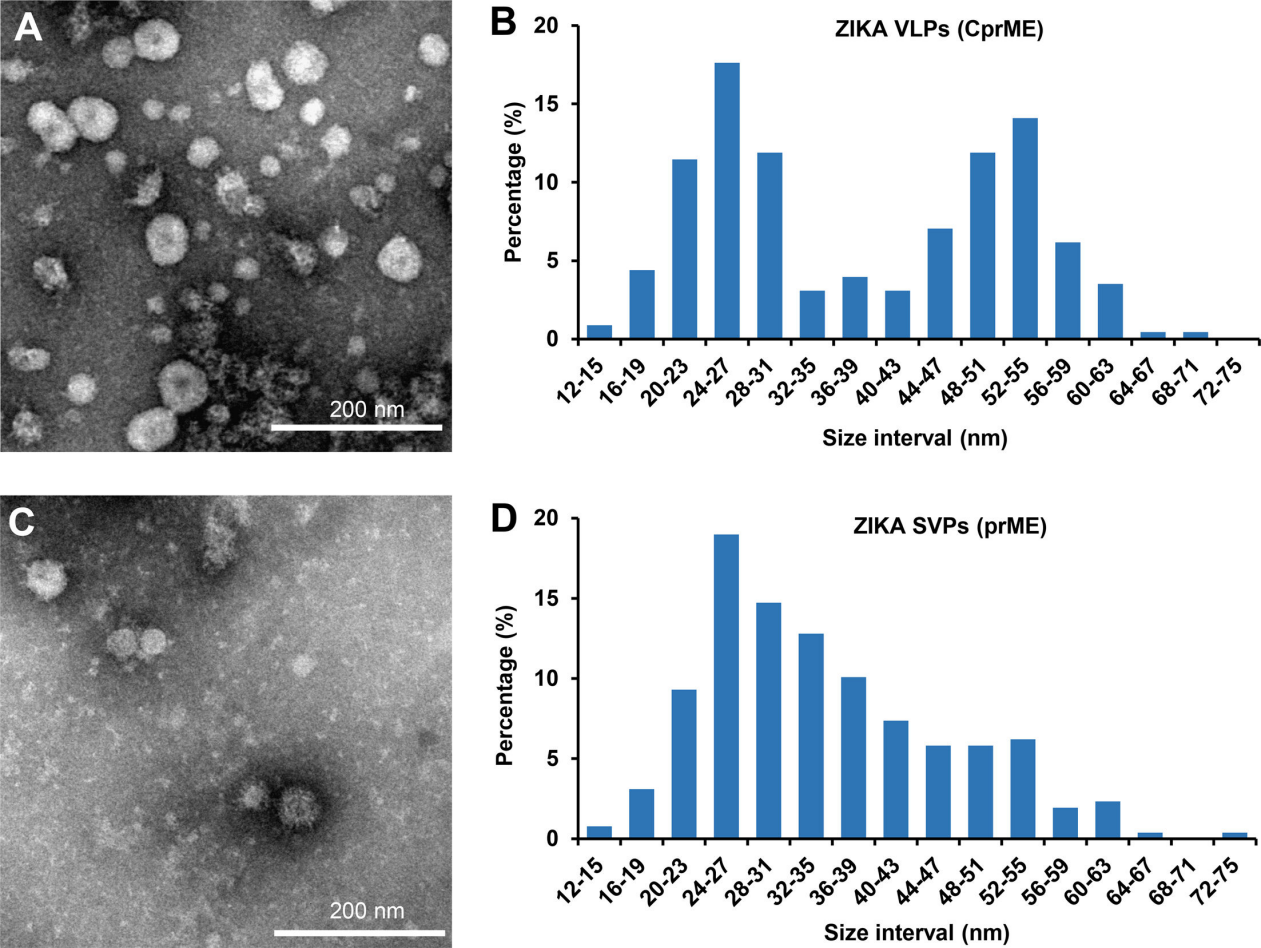
Electron microscopy analysis of Zika VLP and SVP vaccines. (**A**) Transmission electron microscopy photo of purified Zika VLP (CprME) vaccine. (**B**) Size distribution of particles in CprME fraction based on diameter measurements of 227 particles. (**C**) Transmission electron microscopy photo of purified Zika SVP (prME) vaccine. (**D**) Size distribution of particles in prME fraction based on diameter measurements of 258 particles.

The purified VLP/SVP vaccines were analyzed by transmission electron microscopy. Spherical particles with a diameter of ∼20–60 nm were observed in the VLP (CprME) preparation, with particle diameter distribution showing segregation into two groups (Fig. 2A). The larger VLPs had a diameter of 52–55 nm (Fig. 2B), which corresponds with the reported size of complete, infectious ZIKV virions. The smaller particles had a diameter of 24–27 nm (Fig. 2B), which correspond to the size of Zika SVPs. Non-infectious SVPs of about ∼20–30 nm in diameter have previously been observed during natural flavivirus infection (61) and after expression of recombinant flavivirus prME (62, 63). The purified SVP (prME) preparation comprised mostly of ∼20–30 nm diameter SVPs, with a smaller fraction of larger particles (Fig. 2C and D).

### Poor protection against challenge after Zika VLP and SVP vaccination

**Fig 3:**
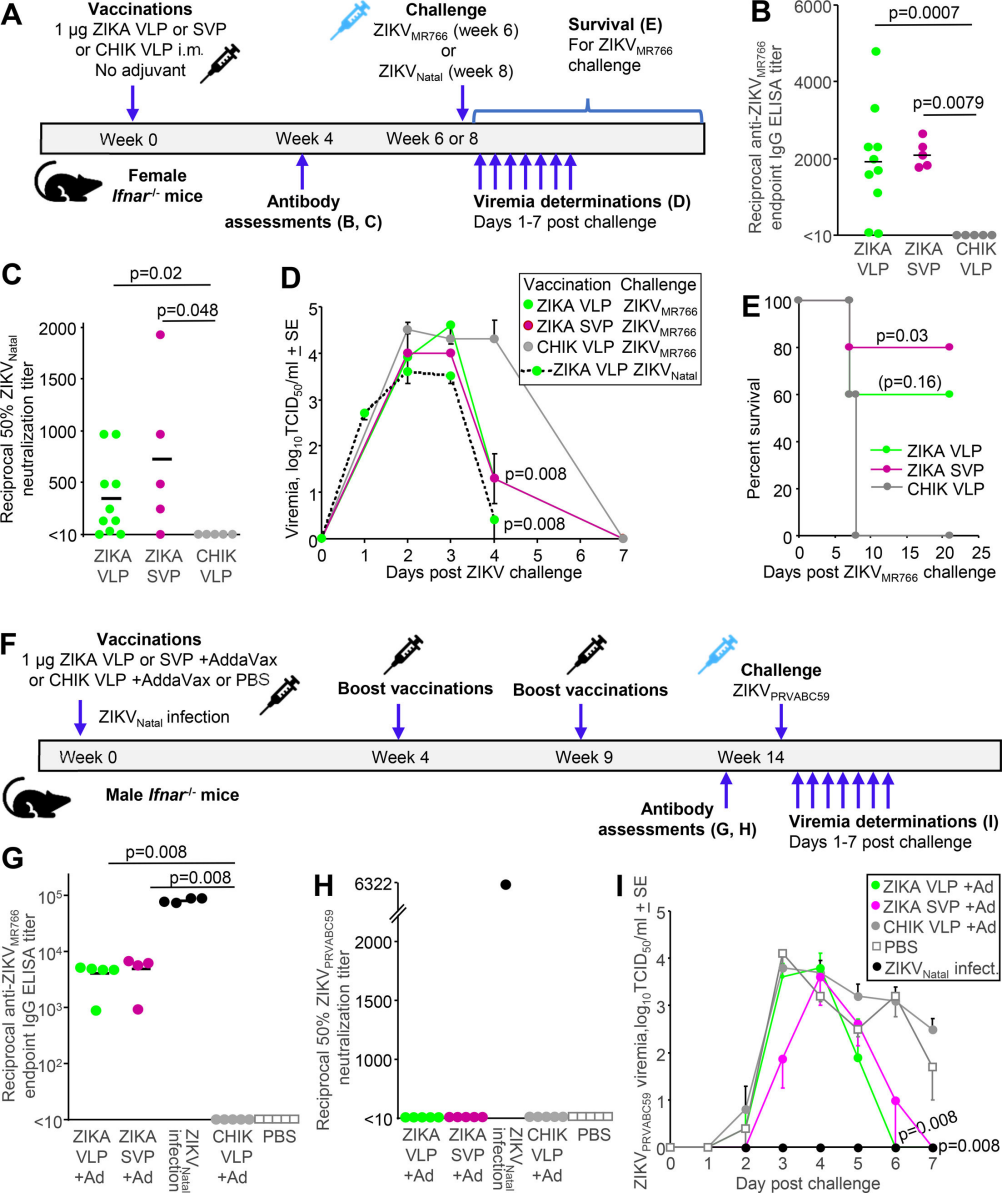
Vaccination of *Ifnar1*^−/−^ mice with Zika VLP or SVP and ZIKV challenge. (**A**) Timeline of vaccination of female *Ifnar1*^−/−^ mice with a single, non-adjuvanted dose of 1 µg Zika VLP or Zika SVP or CHIK VLP (negative control), followed by antibody measurements, and challenge with ZIKV_Natal_ followed by viremia determinations, or challenge with ZIKV_MR766_ followed by viremia and survival determinations. (**B**) ZIKV_MR766_ end point IgG ELISA titers in serum from female *Ifnar*^−/−^ mice after immunization with one dose of the indicated vaccine. Limit of detection was one in 10 serum dilution. (**C**) ZIKV_Natal_ 50% neutralization titers in serum from female *Ifnar1*^−/−^ mice vaccinated as in B. Limit of detection is one in 10 serum dilution. Statistics by Kolmogorov-Smirnov exact tests. (D) Mean viremias post ZIKV challenge (*n* = 5 per group). The limit of detection per mouse was 2 log_10_TCID_50_/mL. Statistics relative to CHIK VLP on day 4. (**E**) Survival of immunized mice after ZIKV_MR766_ challenge. Animals were euthanized when ethically defined end points had been reached. Statistics by log-rank tests relative to CHIK VLP. (**F**) Timeline of vaccination of male *Ifnar1*^−/−^ mice with three 1 µg doses of Zika VLP or SVP, or CHIK VLP (negative control) adjuvanted with AddaVax, or PBS (negative control), followed by serum antibody and viremia determinations after challenge with ZIKV_PRVABC59_. (**G**) Serum ZIKV_MR766_ end point IgG ELISA titers after three vaccinations or after infection with ZIKV_Natal_ (positive control). Limit of detection was one in 10 serum dilution. (**H**) Reciprocal anti-ZIKV_PRVABC59_ 50% neutralization titers. Limit of detection was one in 10 serum dilution. (**I**) Mean ZIKV_PRVABC59_ viremias post challenge (*n* = 4–5 per group). Statistics relative to CHIK VLP on day 6 (for Zika VLP) and day 7 (for Zika SVP). Statistics Smirnov exact tests was used for data in panels B, C, D, G, and I.

The Zika VLP (CprME) and SVP (prME) experimental vaccines were produced at larger scale using suspension Sf9 insect cells. Female *Ifnar1*^−/−^ mice received one dose of 1 µg of the purified VLPs (10 mice) or SVPs (five mice), and antibody responses and protection against ZIKV_Natal_ and ZIKV_MR766_ challenge were assessed (Fig. 3A). As a negative control, five mice were vaccinated with purified chikungunya virus (CHIKV) VLPs (43). Four weeks post VLP/SVP vaccination, significant ZIKV-specific ELISA titers were generated, whereas no ZIKV-specific antibodies were detected after immunization with CHIKV VLPs (Fig. 3B). Most VLP/SVP-vaccinated mice developed significant neutralizing antibody titers against ZIKV, whereas CHIKV VLPs did not induce detectable neutralizing antibody responses (Fig. 3C).

Mice were challenged with the African ZIKV_MR766_ isolate, which is lethal in this model (58), or with the Brazilian ZIKV_Natal_ isolate, which is generally nonlethal in this model (47). Viremias were not significantly suppressed in VLP/SVP vaccinated mice, except on day 4 post challenge (Fig. 3D). Zika SVP-vaccinated mice were nevertheless significantly protected against weight loss that reached ethically defined end points (>20%) that required euthanasia (Fig. 3E). In addition, 60% of mice vaccinated with VLPs survived, although this did not reach statistical significance (Fig. 3E, *P* = 0.16). VLP/SVP vaccination thus provided limited protection against challenge.

Next, we vaccinated mice at three different times (Fig. 3F) with the VLP/SVP vaccines and included an adjuvant, AddaVax, a squalene-based, oil-in-water, nano-emulsion adjuvant formulation similar to the MF59 adjuvant licensed for use in humans (64). Although higher ELISA titers were achieved after three vaccinations (Fig. 3G), no neutralization titers were detected (Fig. 3H). After challenge with ZIKV_PRVABC59,_ VLP/SVP-vaccinated mice again showed only a significant reduction late in the viremic period (Fig. 3I, 6/7 dpi). The results suggested that although three doses and adjuvant increased the ELISA titers, they did not improve protection.

The surprisingly low neutralizing antibody responses (Fig. 3H) suggested poor presentation to the immune system of authentic tertiary and/or quaternary structures, which are deemed important for generation of effective neutralizing antibody responses (19). This may have arisen due to pH issues during production (see below) affecting the vaccine batch used for Fig. 3F to I more than the vaccine batch used for Fig. 3A to E. Alternatively, AddaVax, which contains the surfactants Span 85 (sorbitan trioleate) and Tween 80 (polyoxyethylene 80/sorbitan monooleate) (65), may have destabilized the VLP/SVPs (66–68). These results suggested that these VLP/SVP vaccines adopted and/or maintained authentic conformations poorly.

### Epitope display analysis

**Fig 4:**
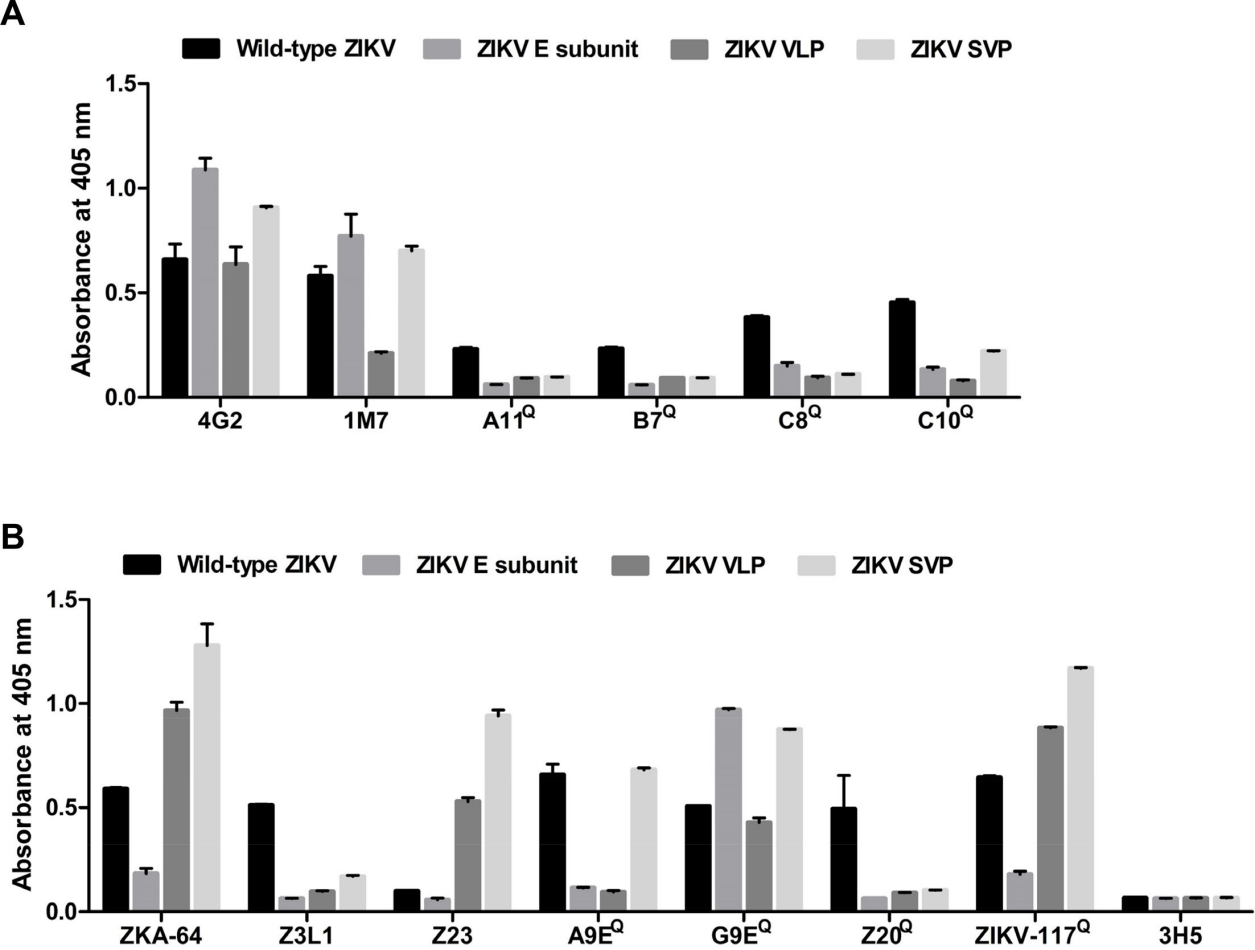
Zika VLP and SVP epitope display analysis. Binding of (**A**) flavivirus cross-reactive anti-E mAbs 4G2, 1M7, A11, B7, C8, and C10 and (**B**) ZIKV-specific anti-E mAbs ZKA-64, Z3L1, Z23, A9E, G9E, Z20, and ZIKV-117 to wild-type ZIKV, ZIKV E subunit, Zika VLP, and Zika SVP vaccines. The mAbs that bind quaternary structure epitopes are marked with “Q”. The DENV2-specific anti-E mAb 3H5 was included as a negative control. The mean of two technical replicates is shown, with error bars indicating the standard deviation.

To explore the tertiary and/or quaternary structures presented by VLP/SVP vaccines, a panel of 14 well-characterized monoclonal antibodies (mAbs) that recognize E protein epitopes on ZIKV (Table 2) were used in a series of ELISAs. The pan-flavivirus mAbs 4G2 and 1M7 (Fig. 4A), which recognize low complexity fusion loop epitopes in domain II of the E, bound with similar efficiency (similar absorbance) to wild-type virus (positive control) and the VLP/SVP vaccines (Fig. 4A). In contrast, the pan-specific flavivirus E dimer epitope (EDE)-dependent mAbs, A11, B7, C8, and C10, bound with relatively lower efficiencies to the VLP/SVP vaccines than they did to wild-type ZIKV and showed absorbance values similar to mAb binding to the largely unstructured E subunit protein (Fig. 4A). The results argue that VLP/SVPs display significantly lower levels of E dimer quaternary epitopes than wild-type virus.

Of the ZIKV-specific mAbs that bind lower complexity protein conformations, Z3L1, ZKA-64, and Z23, the latter two actually bound VLP/SVPs better than wild-type ZIKV (Fig. 4B). Of the remaining mAbs that bound quaternary epitopes (A9E, G9E, Z20, and ZIKV-117), Z20 failed to bind either SVPs or VLPs, and A9E failed to bind VLPs (Fig. 4B).

These results (Fig. 4) illustrated that the quaternary arrangements and/or conformations of E proteins were markedly different between wild-type ZIKV and the VLP/SVP vaccines. This in turn likely explains their limited abilities to protect mice against challenge (Fig. 3D, I). We chose to pursue further SVPs as the vaccine modality of choice in this setting, as 1M7 and A9E effectively recognized SVPs and wild-type ZIKV, but not VLPs (Fig. 4A and B). Similar prME particles have also been shown to be efficacious in dengue virus vaccine design (69), and prME has emerged as the immunogen of choice for many flaviviral vaccines (19).

### Production of stabilized SVP-A264C vaccine in insect cells

**Fig 5:**
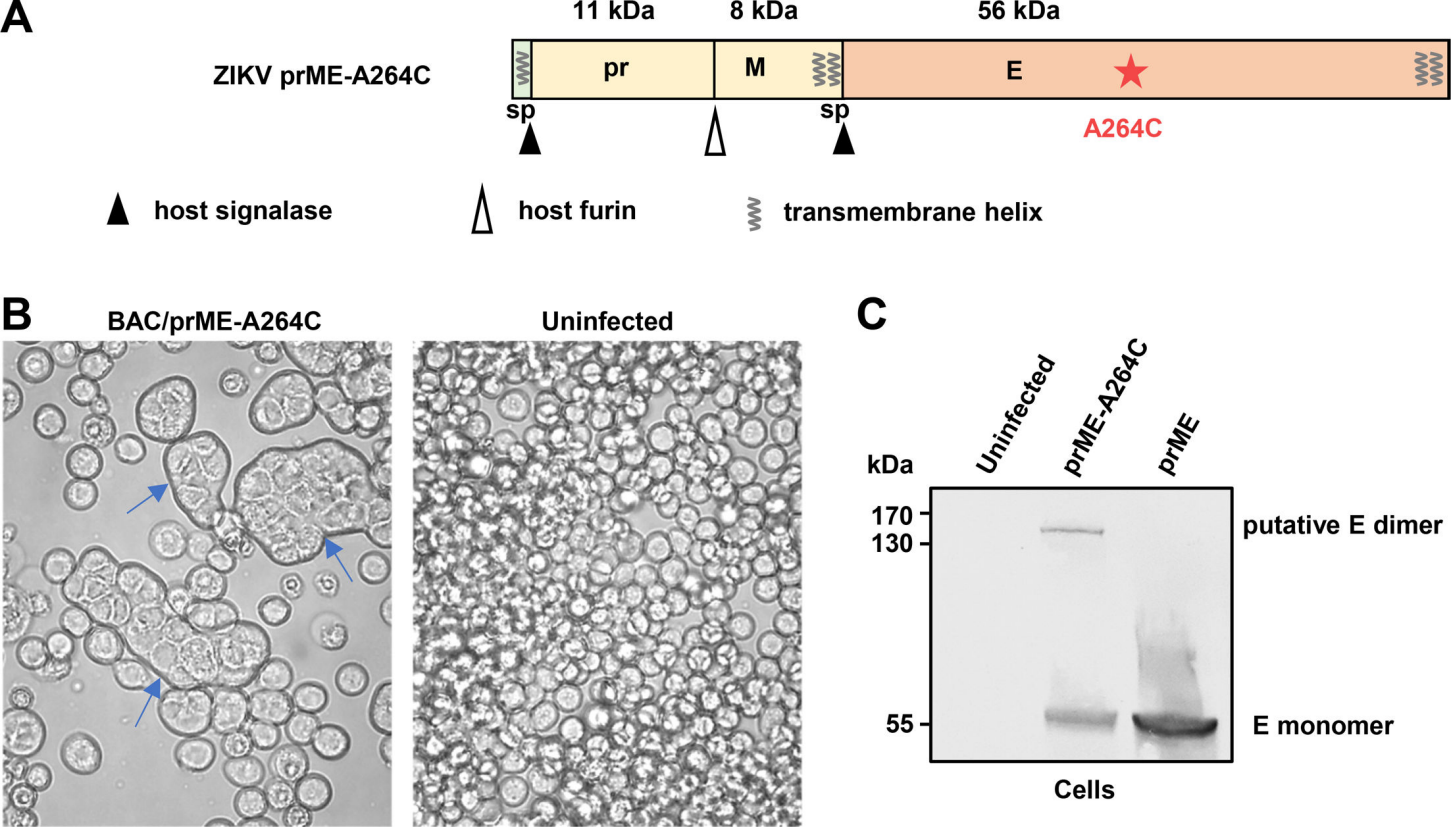
Production of the SVP-A264C vaccine in Sf9 cells. (**A**) Schematic overview of the ZIKV prME structural cassette with the alanine to cysteine (A264C) substitution that promotes covalent linkage of E proteins to produce SVPs with stabilized E homodimers. The molecular mass of each viral protein is shown in kDa. Cleavage sites of host signalase and host furin are indicated, as well as predicted signal peptide (sp) sequences and transmembrane helices. (**B**) Sf9 insect cells infected with indicated baculovirus at 3 days post infection or uninfected cells. Syncytia formations are clearly evident (arrows). (**C**) Sf9 insect cells infected with recombinant baculoviruses expressing the indicated cassettes analyzed by Western blot using pan-flavivirus anti-E mAb 4G2.

Previous studies on DENV and ZIKV showed that displaying stable E homodimers in vaccine formulations can be challenging to achieve, but that covalent linkage of the E proteins within a dimer can improve vaccine efficacy (37, 38, 40, 41, 70). An alanine to cysteine codon substitution (A264C) was thus introduced in the E domain II region of ZIKV prME (Fig. 5A). This mutation allows for a stable antiparallel dimer of E (36). The ZIKV prME-A264C structural cassette (Fig. 5A) was then used to generate the recombinant baculovirus BACe56/ZIKV prME-A264C vaccine construct. Cells expressing ZIKV prME-A264C formed large syncytia (Fig. 5B), similar to cells expressing ZIKV prME (Fig. 1B), arguing that the mutated ZIKV E protein was capable of fusogenic activity and that transition from the prefusion conformation to the fusogenic structure had occurred for at least some of the E proteins (see below).

Infected insect cells were subjected to nonreducing Western blot analysis using the E protein-specific mAb, 4G2. Cells expressing ZIKV prME-A264C showed the expected E monomer band at ∼55 kDa, similar to cells expressing ZIKV prME (Fig. 5C). However, ZIKV prME-A264C expression also led to an additional band of higher molecular weight, likely representing the covalently linked E dimers (Fig. 5C).

### Production and characterization of SVP-A264C vaccine at neutral-pH-adapted insect cells

The VLP/SVP productions described above occurred using culture medium at pH 6.2–6.4, which is the pH range commonly used in baculovirus-insect cell expression systems. However, it is also the pH range at which flavivirus E protein-mediated fusion occurs (71–73). The latter results in an irreversible conformational transition of the prefusion ZIKV E protein dimers into a trimeric state to expose the fusion loop and initiate membrane fusion. The syncytia formation seen during SVP/VLP production (Fig. 1B and 5B) argues that fusogenic activity of the ZIKV E protein had been triggered by the low pH of the insect cell culture medium. We reasoned that presenting the immune system with SVPs whose E proteins had largely undergone the transition to a fusogenic structure would result in reduced induction of protective antibody responses, as the latter generally requires presentation of envelope proteins in their prefusion conformation (19, 74, 75). We thus sought to produce SVP vaccines at pH 7.0, which is above the threshold for flavivirus E protein-mediated fusion (71, 72).

**Fig 6:**
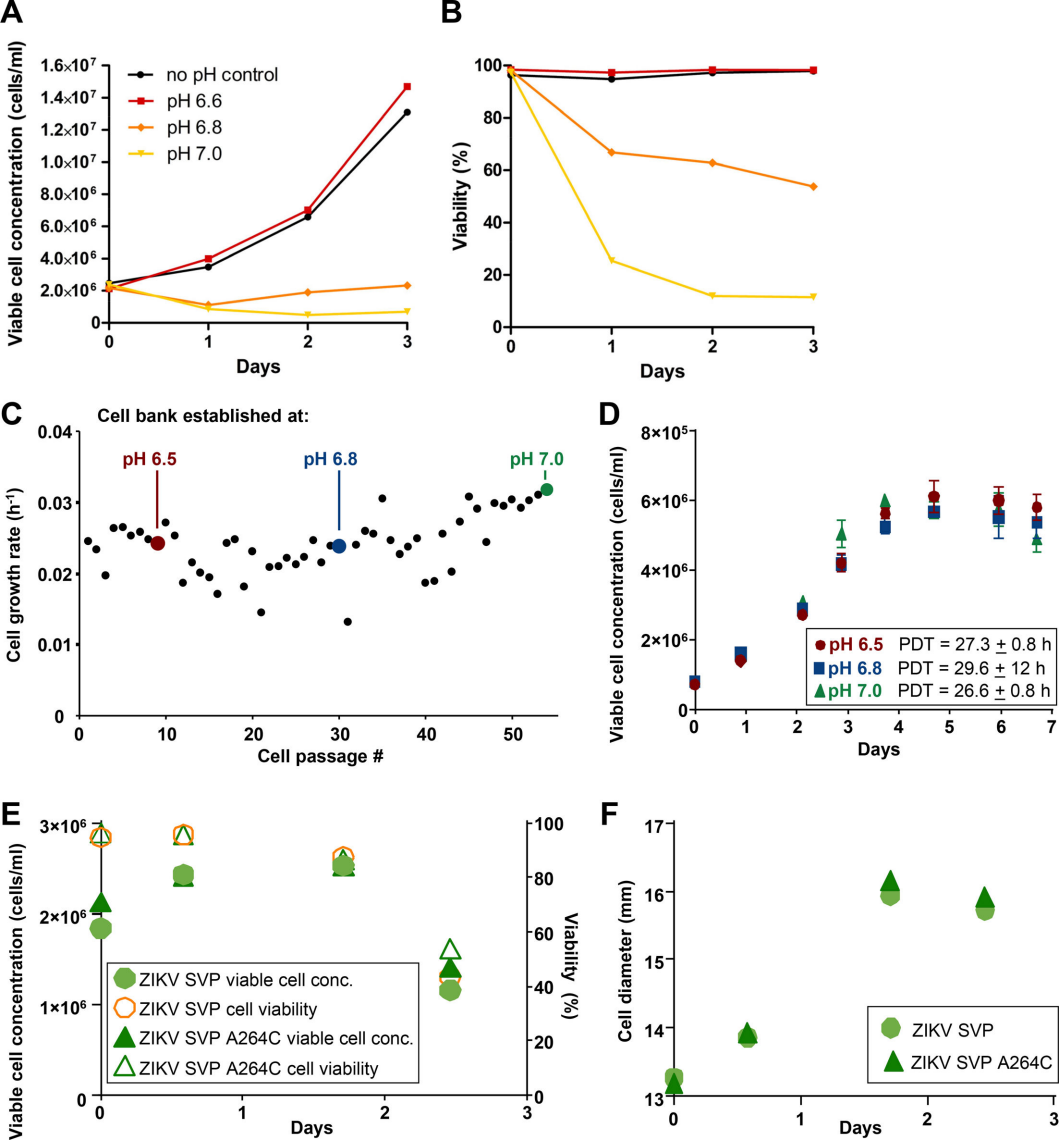
Production of ZIKV SVP and SVP-A264C vaccines at neutral pH. For panels **A–B**, Sf9 cells (cell density at day 0: 2 × 10^6^ cells/mL) were grown at standard medium pH (no pH control) or at pH 6.6, pH 6.8, and pH 7.0 for 3 days. (**A**) Viable cell concentration. (**B**) Cell viability. For panels **C–F**, production of ZIKV SVP and SVP-A264C using Sf9 cells adapted to neutral pH. (**C**) Cell growth rate during adaptation of insect Sf9 cells to neutral pH via adaptive laboratory evolution. At passage #1, culture pH was changed from standard (≈6.2) to 6.5. After establishment of each cell bank, cell culture pH was changed to the next pH iteration. (**D**) Cell growth kinetics of new high pH-adapted cell lines. PDT, population doubling time. (**E**) Cell growth and viability kinetics and (**F**) cell diameter during production of SVP and SVP-A264C vaccines using pH 7-adapted insect Sf9 cells.

To investigate whether suspension Sf9 insect cells would tolerate being cultured at higher pH, uninfected cells were grown for 3 days with no pH control (i.e., standard culture conditions at pH ≈ 6.2) and at pH 6.6, pH 6.8, or pH 7.0, and cell concentration and cell viability were measured daily. The cells cultured in medium without pH control (for which the pH gradually dropped from 6.2 to 6.0 during the experiment) as well as the cells cultured in medium of pH 6.6 grew to cell densities of 10^7^ cells/mL (Fig. 6A) and showed high cell viability (Fig. 6B). In contrast, growth of cells at pH 6.8 and pH 7.0 was significantly slower (Fig. 6A), with low cell viability (Fig. 6B). Sf9 insect cells were thus adapted to neutral pH via adaptive laboratory evolution using a step-wise approach as described previously for another insect cell line (35). Briefly, Sf9 cells which are typically cultured at a standard pH of ≈6.2 were subcultured at pH 6.5, 6.8, and 7.0 until a growth rate between 0.02 and 0.03 divisions/hour was achieved for ≥5 passages (Fig. 6C). At this point, cells were considered adapted, and a cell bank was established. Growth kinetics of cells adapted to higher pH by adaptive laboratory evolution showed population doubling times within the expected range for standard, non-adapted Sf9 cells (≈ 24–28 hours) (Fig. 6D).

ZIKV SVP and SVP-A264C vaccines were produced using Sf9 cells adapted to neutral pH (7.0) at 2 L bioreactor scale with constant maintenance of culture conditions at pH 7.0. Infection kinetics of adapted cells producing SVP and SVP-A264C were typical of a process using the MOI herein employed (2 TCID_50_ units per cell), i.e., minimal cell growth after infection and onset of cell viability drop after 24 hours (Fig. 6E) as well as increase in cell diameter (Fig. 6F) in line with the prior infection experiments. As controls, both vaccines were also produced using non-adapted Sf9 cells at pH 6.

**Fig 7:**
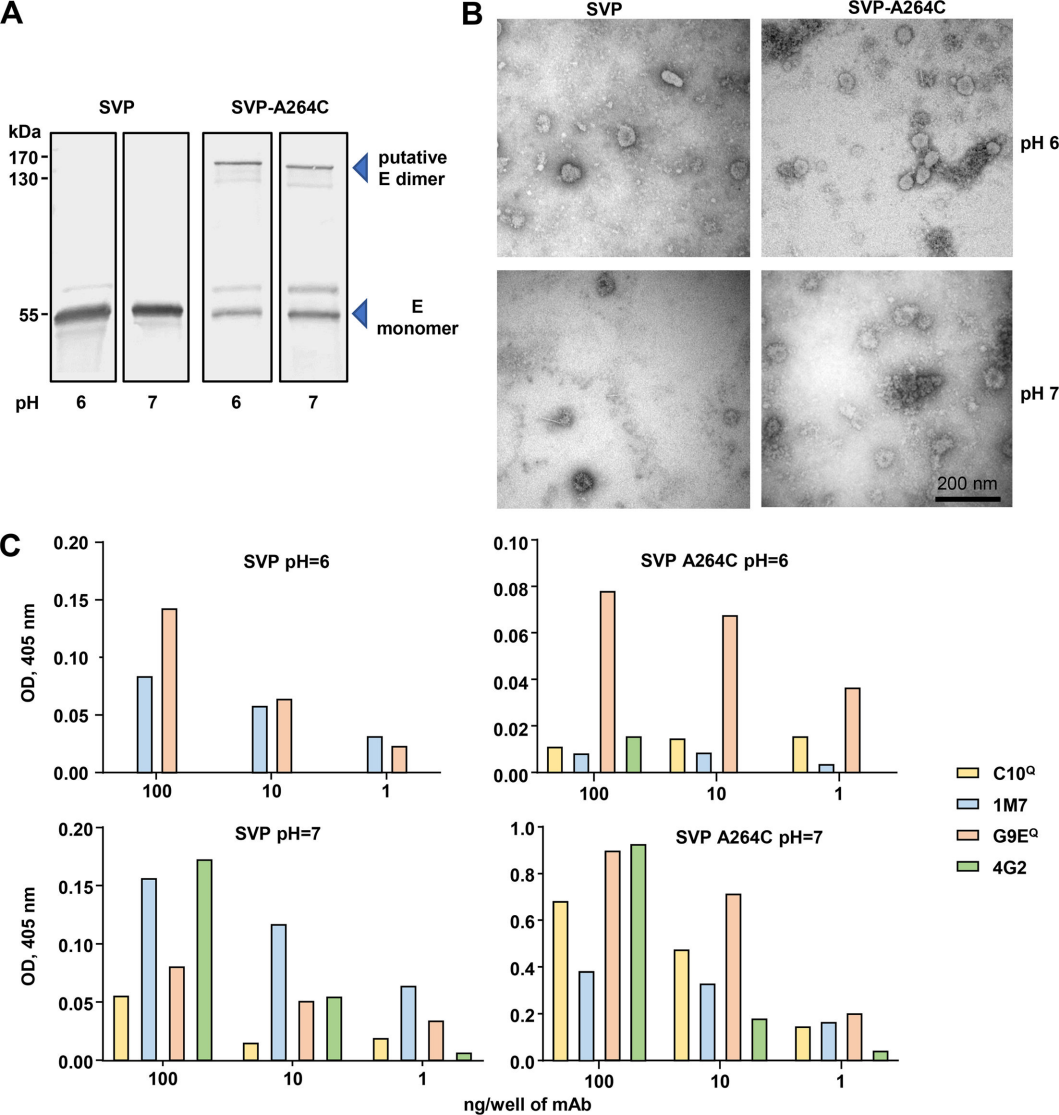
Characterization of ZIKV SVP and SVP-A264C vaccines produced at pH 7 or pH 6. (**A**) SVP-A264C and SVP vaccines were produced at pH 7 or pH 6, sucrose gradient purified, and analyzed by Western blotting using the anti-E mAb 4G2. (**B**) Transmission electron microscopy photos of the vaccines in A. (**C**) Epitope display analysis of SVP-A264C and SVP vaccines produced at pH 6 or pH 7. Binding of flavivirus cross-reactive anti-E mAbs 4G2, 1M7, and C10 and ZIKV-specific anti-E mAb G9E. C10 and G9E bind quaternary structure epitopes.

Western bot analysis of purified SVP and SVP-A264C vaccines produced at pH 6 and 7 illustrated the putative covalently linked dimer for the SVP-A264C vaccine and the E monomers for both vaccines produced at either pH (Fig. 7A). Spherical particles of ∼20–60 nm in diameter were observed in the purified samples by electron microscopy (Fig. 7B), indicating that prME-A264C expression resulted in SVP production and that particles could also be formed at pH 7.

To investigate epitope presentation, the binding of a select panel of mAbs (Table 2) to E protein epitopes was measured by ELISA (as in Fig. 4). The highest level of binding across all four mAbs, with C10 and G9E recognizing quaternary epitopes, was seen for the SVP-A254C vaccine produced at pH 7 (Fig. 7C).

### Evaluation of the SVP-A264C vaccine in a murine ZIKV challenge model

**Fig 8:**
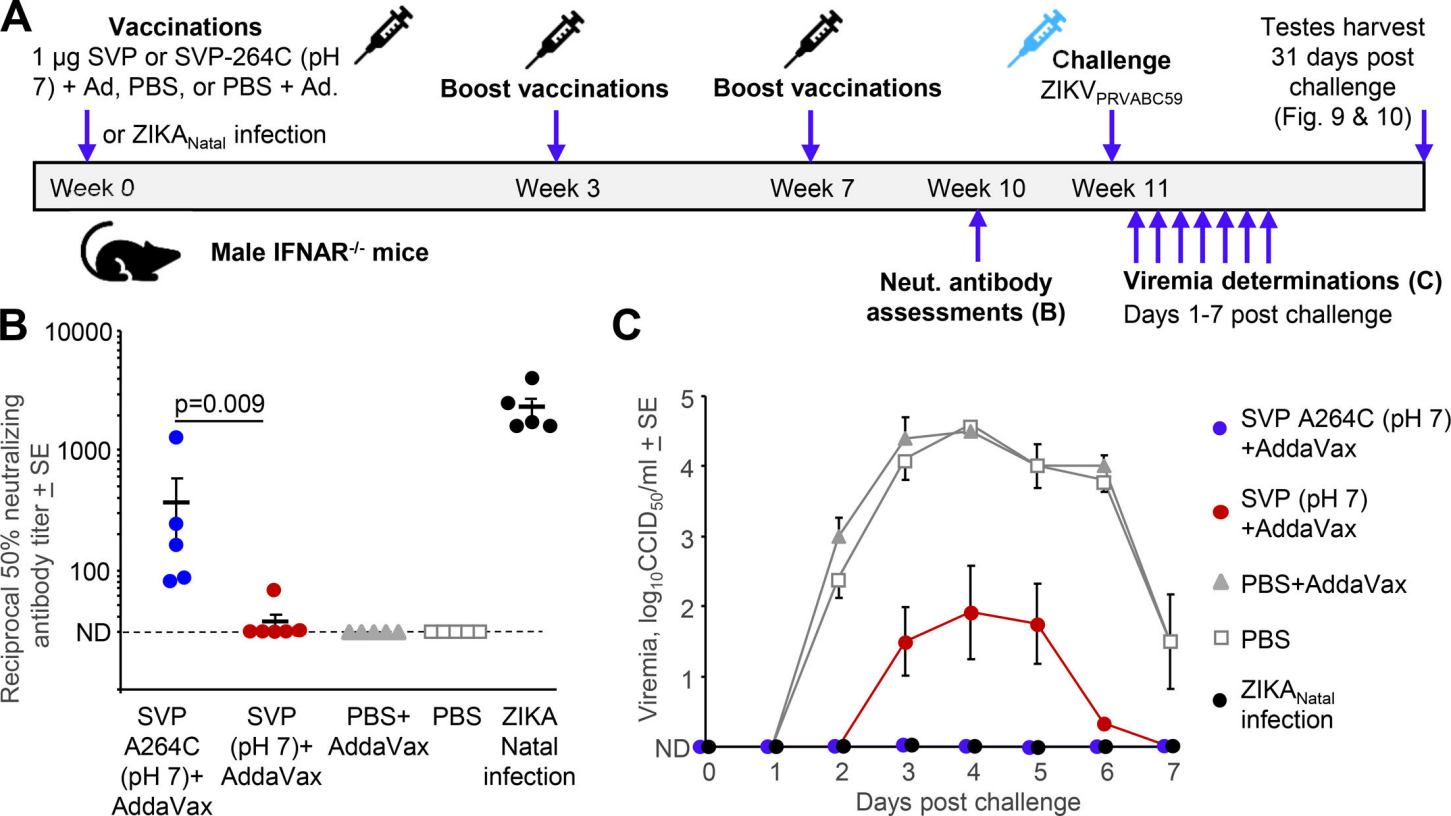
Vaccination and challenge study with the SVP-A264C vaccine produced at pH 7. (**A**) Timeline of three 1 µg intramuscular vaccinations of male *Ifnar1*^−/−^ mice with SVP-A264C or SVP vaccines produced at pH 7.0. The vaccines were adjuvanted with AddaVax. Negative control mice received PBS with AddaVax or PBS. Mice recovered from ZIKV_Natal_ infection (10^4^ TCID_50_ s.c. week 0) represented positive controls; ZIKV_Natal_ infection is nonlethal in this setting. Sera was collected prior to challenge to determine neutralizing antibody titers, with serum viremias determined days 1–7 post challenge with ZIKV_PRVABC59_ (10^3^ TCID_50_ s.c.). Mice were euthanized on day 31 and testes harvested (see Fig. 9 and 10). (**B**) Mean and individual serum ZIKV_PRVABC59_ 50% neutralization titers from mice that had received the indicated vaccines, PBS controls, or after infection with ZIKV_Natal_. Limit of detection was 1 in 30 dilution of serum (dotted line; data points plotted on this line represent not detected, ND). Statistics by Kolmogorov-Smirnov exact test. (**C**) Mean ZIKV_PRVABC59_ viremias post challenge for the same groups as in B (*n* = 5–6 mice per group).

Male *Ifnar1*^−/−^ mice were vaccinated at three different times (Fig. 8A) with 1 µg of AddaVax-adjuvanted SVP-A264C vaccine or SVP vaccine, both produced at pH 7 (Fig. 8A). Mice receiving PBS or PBS + adjuvant served as negative controls, and mice recovered from a ZIKV_Natal_ infection served as a positive control group. Mice vaccinated with 1 µg of the SVP-A264C (pH 7) vaccine developed statistically significant higher neutralization titers than mice vaccinated with 1 µg of the SVP (pH 7) vaccine (*P* = 0.009), although neutralization titers for SVP-A264C (pH 7) vaccination were ≈ 6 fold lower than those generated by ZIKV_Natal_ infection (Fig. 8B). No detectable neutralization titers were seen after PBS inoculation (Fig. 8B).

After challenge with ZIKV_PRVABC59_, the negative control PBS groups showed the expected viremias, whereas SVP-A264C (pH 7) vaccinated mice showed no detectable viremia on any day (limit of detection was 2 log_10_CCID_50_/mL) (Fig. 8C). SVP (pH 7)-vaccinated mice showed viremia levels that were reduced by ≈ 2.5–3 logs on 2–6 dpi when compared to PBS controls (Fig. 8C). Thus, both the A264C substitution and production at pH 7 substantially improved the ability of these SVP vaccines to mediate protection against viremia.

### Immunization with SVP-A264C (pH 7) vaccine protected mice from testicular damage

**Fig 9:**
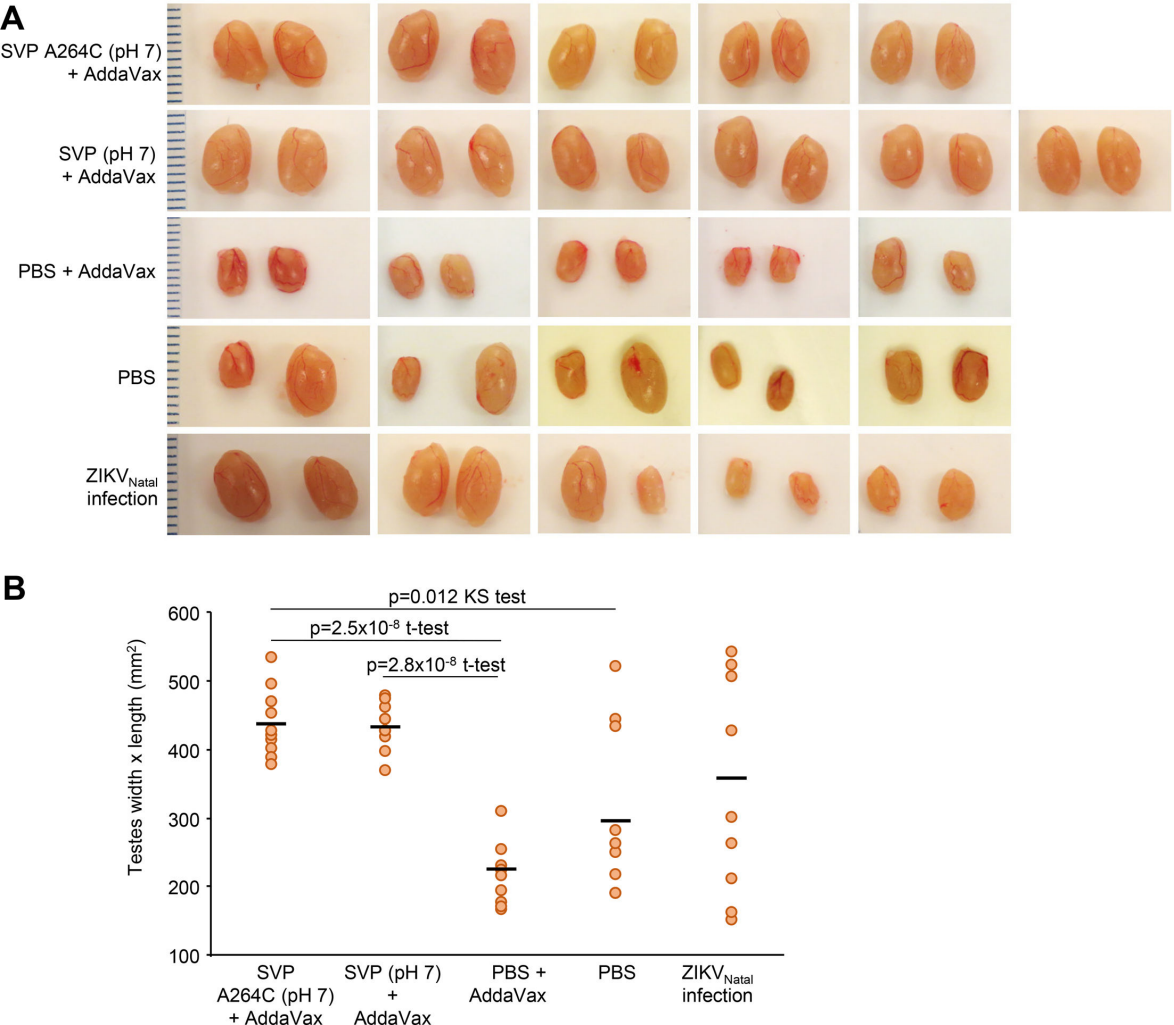
Testes images and sizes after ZIKV_PRVABC59_ challenge. (**A**) For the groups of male *Ifnar1*^−/−^ mice described in Fig. 8, series of five representative photographs of testes harvested at 31 days post challenge with ZIKV_PRVABC59_. Ruler on the left showing 1 mm increments. (**B**) Dimensions of the testes shown in A. Bars represent means. Statistics by *t*-tests or Kolmogorov-Smirnov exact test (KS).

ZIKV_PRVABC59_ infection of male *Ifnar1*^−/−^ mice results in overt reduction in testis size and marked histopathological changes characterized by destruction of seminiferous tubules (24, 45). Thus, as expected, the PBS control groups showed clear reductions in testes size (Fig. 9A and B). Testis size reductions were also observed in mice with past ZIKV_Natal_ infections, with these size reductions probably due to ZIKV_Natal_ infection, rather than ZIKV_PRVABC59_ challenge. Importantly, mice that had been immunized with adjuvanted SVP-A264C (pH 7) vaccine or SVP (pH 7) vaccines showed normal testis size after challenge (Fig. 9A and B).

**Fig 10:**
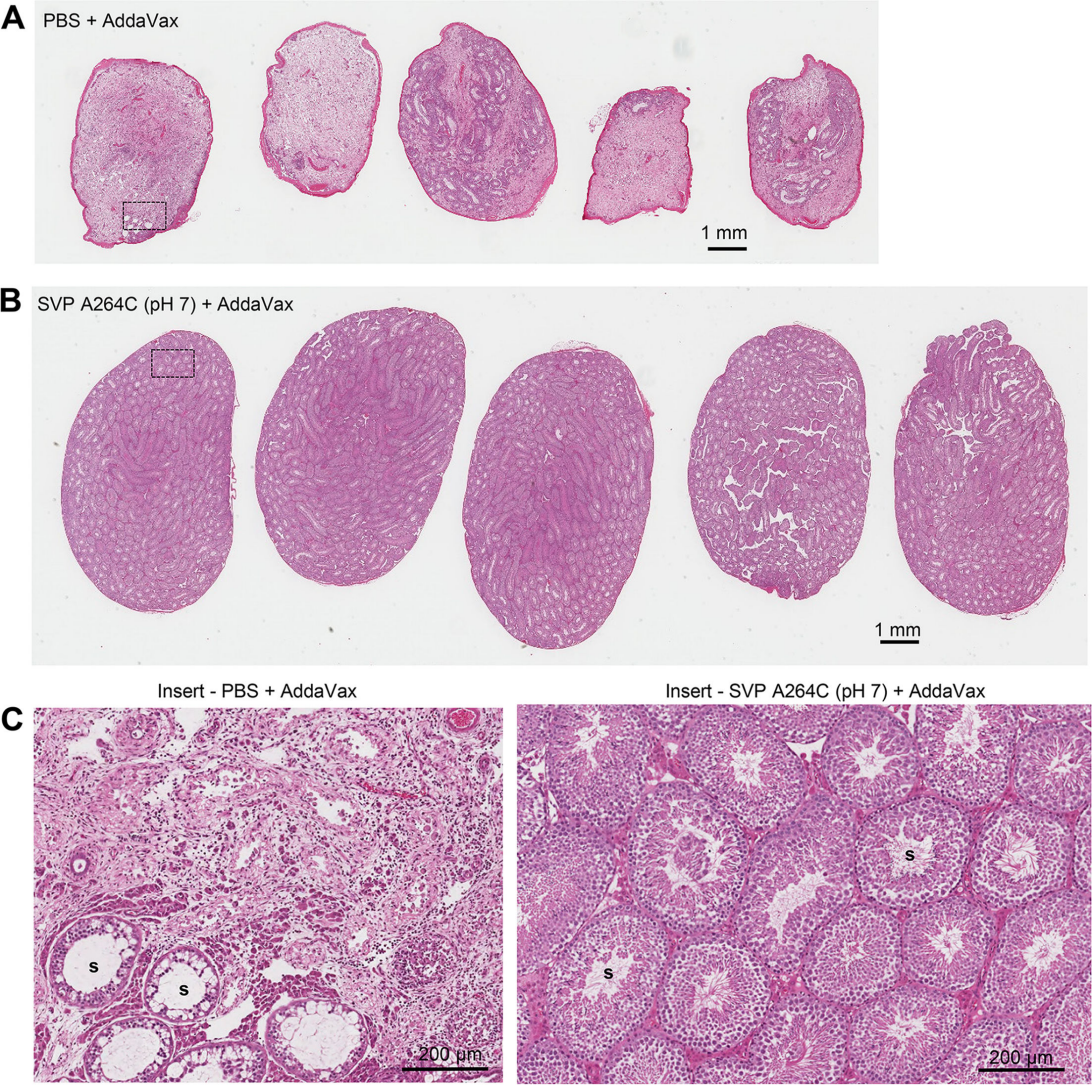
Histopathology of testes after ZIKV_PRVABC59_ challenge. (**A**) H&E-stained sections of testes from mice that received PBS + adjuvant (negative control). Testes were harvested 31 days after challenge with ZIKV_PRVABC59_. (**B**) As for A, but for mice that were vaccinated with SVP-A264C (pH 7) + AddaVax (same mice as described in Fig. 8 and 9). (**C**) Enlargements of A and B showing overt loss of seminiferous tubules or disruption of seminiferous tubule architecture. The SVP-A264C (pH 7) + AddaVax vaccine group shows normal seminiferous tubule morphology. S, lumen of the seminiferous tubules.

H&E staining of testes from the PBS + AddaVax control group illustrated the reduced size and the previously described (24, 45) loss and disruption of seminiferous tubule architecture (Fig. 10A and C). No such testicular damage was seen in mice vaccinated with the SVP-A264C (pH 7) vaccine (Fig. 10B and C).

## DISCUSSION

Herein, we describe the generation of a baculovirus vaccine comprising prME proteins of ZIKV, with both an envelope protein substitution, A264C, that stabilizes E dimer formation (37, 38, 40, 41, 70) and SVP production at pH 7 to maintain the vaccine structure in the prefusion conformation (74, 75). The SVP A264C (pH 7) vaccine protected mice from viremia against ZIKV challenge, whereas VLP/SVP vaccines, without the A264C substitution and produced at the conventional lower pH, provided only limited protection. The SVP A264C displayed a higher portion of E dimers, although dimerization was not complete. Further efforts to enhance immunogenicity may involve additional engineering of the ZIKV E glycoprotein.

The study highlights the importance of challenge experiments to ascertain whether the responses induced by an experimental vaccine actually mediate protection against virus challenge (76). The study also reiterates the contention that effective flavivirus vaccines need to present the immunogen in an authentic tertiary and quaternary structure with a prefusion conformation (19).

Effective ZIKV prME (SVP) vaccines with comparable results to those presented herein have been developed using mammalian cell production systems (70, 77, 78). For example, three vaccinations of *Ifnar1*^−/−^ mice with 10 µg of a A264C prME ZIKV vaccine adjuvanted with alum and monophosphoryl lipid A prevented viremia in most mice (70), and two vaccinations of *Ifnar1*^−/−^ mice with 10 ug of a A264C prME ZIKV vaccine adjuvanted with alum reduced the RNAemia to undetectable levels in 3/10 mice. The pH range of mammalian cell culture fluid is usually 7.0–7.2, which would likely result in retention of the prefusion conformation in these vaccines. Mammalian systems would thus not require pH adaptation; however, mammalian production systems remain expensive, yields can be low, and scale-up can be difficult (79, 80). A recent alternative method for generating VLP-like ZIKV vaccines is the use of a chimeric virus, comprising an insect-specific virus (Binjari virus) backbone and prME from ZIKV (21, 24, 56). A single 2 µg dose of unadjuvanted chimeric vaccine completely protected *Ifnar1*^−/−^ mice from viremia and testes damage (24). This potentially highlights an advantage of CprME over prME particle vaccines, with the Binjari/Zika-prME chimera folding into authentic ~50 nm virion particles (24). Cleavage of C from CprME is achieved by the NS2B/NS3 protease in infected cells, with Binjari virus NS2B/NS3 able to correctly cleave Binjari virus capsid from ZIKV prME (24). Whether the baculovirus system can provide C-prME cleavage at the correct site remains unclear and may warrant further investigations. However, other factors may be in play to explain the efficacy of the Binjari chimera, such as immunopotentiating impurities in the vaccine preparations, glycosylation patterns (79), and/or some kind of limited abortive RNA replication (81). Perhaps useful to note is that effective ZIKV mRNA vaccines also encode just prME (18, 19), with mammalian cells generally unable to mediate cleavage of capsid from CprME. Perhaps an overriding consideration is that baculovirus systems have been approved for manufacture of human vaccines (82), whereas mosquito cell lines (currently used for chimeric Binjari vaccines [83]) have yet to pass this hurdle. Additional advantages of baculovirus expression of glycoproteins in insect cells are the established history of safety and industrial applications (>40 years) (84), the predictable and homogeneous glycosylation patterns, and the compatibility with expression of arboviral proteins and VLPs (85).

Our study has a number of limitations; firstly, we have not explored the full range of adjuvants that are currently available and that may be suitable for Zika VLP/SVP vaccines. This includes alum (86, 87) and ASO series adjuvants (88, 89), as well as promising adjuvants yet to be approved for use in humans (90, 91). Secondly, although, for instance, dengue virus VLP studies in nonhuman primates have also used up to three vaccinations (92), strategies to reduce the requirement for multiple vaccinations are desirable for such vaccines, especially in resource-poor settings where they are usually most needed. A single vaccination with ensuing lifelong immunity (93) remains a laudable goal. Lastly, side-by-side comparisons of any new vaccine with the latest in mRNA vaccine development (18) are likely to be warranted (94). Such studies might include evaluation of the longevity of responses and analysis of IgG isotype profiles, areas where mRNA vaccine performance may be suboptimal (22, 95).

In conclusion, we have illustrated a development path for the generation of a baculovirus-derived Zika SVP vaccine, which has required both a mutation to stabilize the E dimers and generation of Sf9 insect cells capable of producing vaccine at pH 7.

## AFTER EPUB

*[This article was published on 27 February 2025 with errors in the affiliation numbers in the byline. The errors were corrected in the current version, posted on 2 April 2025.]*
